# Genetic variants in the MicroRNA biosynthetic pathway Gemin3 and Gemin4 are associated with a risk of cancer: a meta-analysis

**DOI:** 10.7717/peerj.1724

**Published:** 2016-03-15

**Authors:** Wenbo Zhu, Jun Zhao, Jieyu He, Daxun Qi, Lina Wang, Xu Ma, Pei Liu

**Affiliations:** 1 Public Health, Southeast University, Nanjing, Jiangsu, China; 2 National Research Institute for Family Planning, National Research Institute for Family Planning, Beijing, China

**Keywords:** MicroRNA biosynthetic pathway, Gemin3, Gemin4, Cancer

## Abstract

The effects of the microRNA (miRNA) processing genes Gemin3 and Gemin4 on cellular signaling pathways could have a major impact on the risk of cancer. Several studies concerning the association between the Gemin3 rs197412, Gemin4 rs7813 and Gemin4 rs2740348 polymorphisms with cancer susceptibility have been published. The present meta-analysis summarized this evidence and evaluated the precision of these relationships. Relevant studies (published prior to December 16th, 2015) without language restriction were identified using the PubMed, Web of Science and China National Knowledge Infrastructure (CNKI) on-line databases. The data were extracted from the eligible studies and were processed using Stata 12.0 software. Seven studies (2,588 cases and 2,549 controls) indicated that the rs7813 polymorphism was significantly associated with increased cancer risk (TT vs TC + CC, OR = 1.18 95% CI [1.05–1.32]). Six studies (1,314 cases and 1,244 controls) indicated that rs2740348 was associated with an increased cancer risk (GG vs. GC + CC, OR = 1.41 95% CI [1.00–1.83]). However the rs197412 polymorphism was not associated with an increased cancer risk (OR = 0.97 95% CI [0.80–1.19]). Our results suggest that the Gemin4 rs7813 T > C and rs2740348 G > C polymorphisms are associated with cancer susceptibility.

## Introduction

Approximately 1,665,540 new cancer cases and 585,720 cancer deaths were projected to occur in the United States in 2014 (Siegel et al., 2014). Cancer is caused by the uncontrolled proliferation and inappropriate survival of damaged cells, as these events lead to tumor formation (Esquela-Kerscher & Slack, 2006).

The incidence of cancer is a process that involves a variety of factors, and abnormal cell signal transduction pathway activity is considered to be one such essential factor. Identifying genetic markers of cancer susceptibility might help to reduce cancer mortality via early diagnosis and personalized therapy (BM, AI & CC, 2010).

MicroRNAs (miRNAs) are a group of small non-coding molecules that can affect the stability of mRNA to induce mRNA cleavage or translational repression (Bartel, 2004). MiRNAs are involved in nearly every biological process (Kim, Han & Siomi, 2009), and emerging studies indicate that abnormal miRNA activities may play an important role in increasing tumorigenesis risk (Esquela-Kerscher & Slack, 2006).

**Figure 1:**
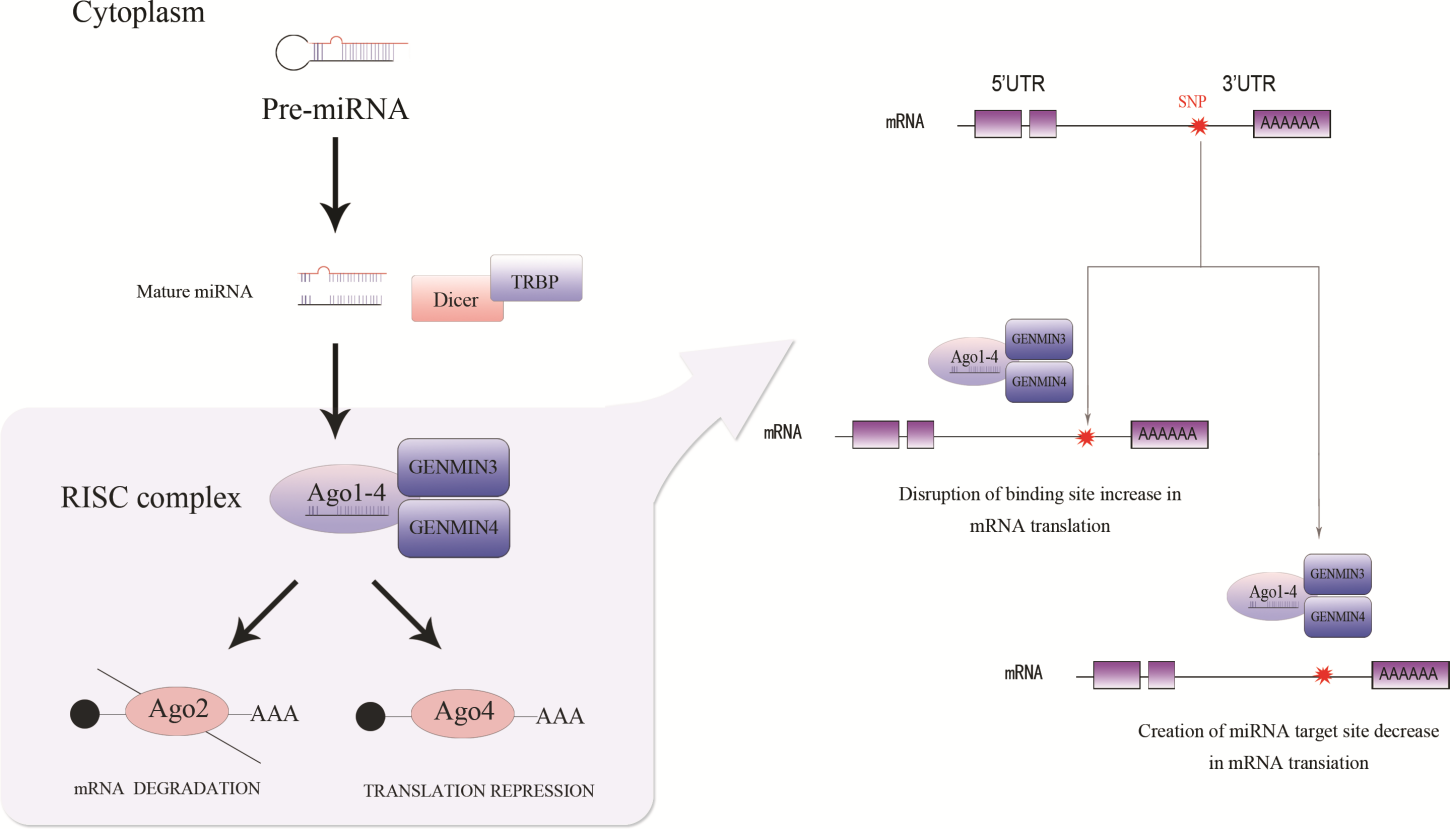
MicroRNA Biosynthetic processing mechanism.

In the biogenesis of miRNAs, the Argonaute proteins (Ago1-4) along with Gemin3 and Gemin4 selectively bind to the guide strand to facilitate the formation of an miRNA-RNA-induced silencing complex (RISC) (Slaby et al., 2012). Single nucleotide polymorphisms (SNPs) may be present in miRNA-binding sites, and mature miRNAs negatively regulate the expression level of their target genes via two distinct mechanisms (Bartel, 2004). In the first mechanism, miRNAs block target gene expression at the translational level with imperfect complementarity. In the second mechanism, miRNAs bind to their mRNA targets with perfect (or nearly perfect) complementarity to induce the RNA-mediated interference pathway (Esquela-Kerscher & Slack, 2006) (Fig. 1). Alterations in the miRNA biosynthesis pathway can lead to global miRNA deregulation. Because miRNAs are involved in a wide range of developmental and physiological processes, deregulation of miRNA processing pathways could potentially impact the transcription and splicing of miRNAs as well as the transcriptional regulation of genes that play fundamental roles in cancers and/or many other human diseases (Kim et al., 2010; Melo & Melo, 2014). Since the impairment of mature miRNAs is emerging as a feature of human cancers (Sonia et al., 2010), given the critical function of Gemin3, Gemin4 and Ago1-4 in miRNA biosynthetic pathway. The host genomic polymorphism of those genes may represent keydeterminants of cancers. SNPs that deregulate miRNAs may alter the expression level of genes related to disease susceptibility (Horikawa et al., 2008; Liu et al., 2012a). Although several studies have investigated the association between the Gemin3 rs197412 T > C, Gemin4 rs7813 T > C and rs2740348 G > C polymorphisms with cancer susceptibility, the results were contradictory and uncertain. Hence, a metaanalysis based on the Preferred Reporting Items for Systematic reviews and Meta-Analyses (PRISMA) criteria (Moher et al., 2009) was imperative to assess the associations between cancer susceptibility and the Gemin3 rs197412, Gemin4 rs7813 and Gemin4 rs2740348 polymorphisms.

## Materials and Methods

### Literature search

Relevant works were identified using the Web of Science, PubMed and CNKI online databases (published prior to December 16th, 2015). We used the following keyword search terms: “cancer or carcinoma,” “tumor or tumour”, “Gemin3, Gemin4, Ago1-4,” “rs197412, rs7813, rs2740348,” and “polymorphism or SNP.”

### Data extraction and quality assessment

The PRISMA guidelines were used as the main criteria in our study, which employed a 27-item checklist and a four-phase flow diagram (S1 PRISMA Checklist) (Moher et al., 2011). We used PROSPERO registrants to compare the planned methods with the final report.

The selection detail of studies for our analysis based on the following criteria: (i) studies that assessed an association between the rs197412, rs7813 and rs2740348 polymorphisms and cancer risks; (ii) studies that contained related casecontrol studies; and (iii) studies that contained available and useful data on genotype frequency for estimating odds ratios (ORs) and 95% confidence intervals (95% CIs). The exclusion criteria included the following: (i) reviews, conference abstracts, or animal studies; (ii) studies lacking sufficient data for a meta-analysis; and (iii) studies reporting data that overlapped with already included studies. Study quality was assessed using the Newcastle-Ottawa Scale (NOS).The NOS scores ranged from 0 to 9, and an NOS score greater than or equal to 6 was considered to indicate a high-quality study.

### Statistical analysis

The ORs and 95% CIs were summarized to evaluate the relevance of each association between the three SNPs and cancer risk using five genetic models: an allele model, a heterozygote model, a homozygote model, a dominant model and a recessive model. *P*-values and *Z* scores were the indices used to evaluate the low-frequency variants in meta-analysis (Evangelou & Ioannidis, 2013). We analyzed these results to enhance their reliability.

We applied Higgins’s (*I*^2^) test for heterogeneity. Generally, if *I*^2^ >50% (Higgins et al., 2003), the random-effects model (Dersimonian & Laird, 1986) was used to evaluate the pooled ORs and the fixed-effects model was applied to everything else.

Data conforming to Hardy-Weinberg equilibrium (HWE) at a significance level of *P* < 0.05 were considered incompatible. If any single study was removed from the analysis or if studies with data poorly conforming to HWE were excluded, sensitivity analysis was used to assess the influence of each study on the pooled OR. We used funnel plots and Begg’s test to evaluate potential publication bias. The significance of these results was evaluated based on an asymmetric plot with a level of significance of *P* < 0.05. The analyses were conducted using Stata 12.0 software.

## Results

**Figure 2:**
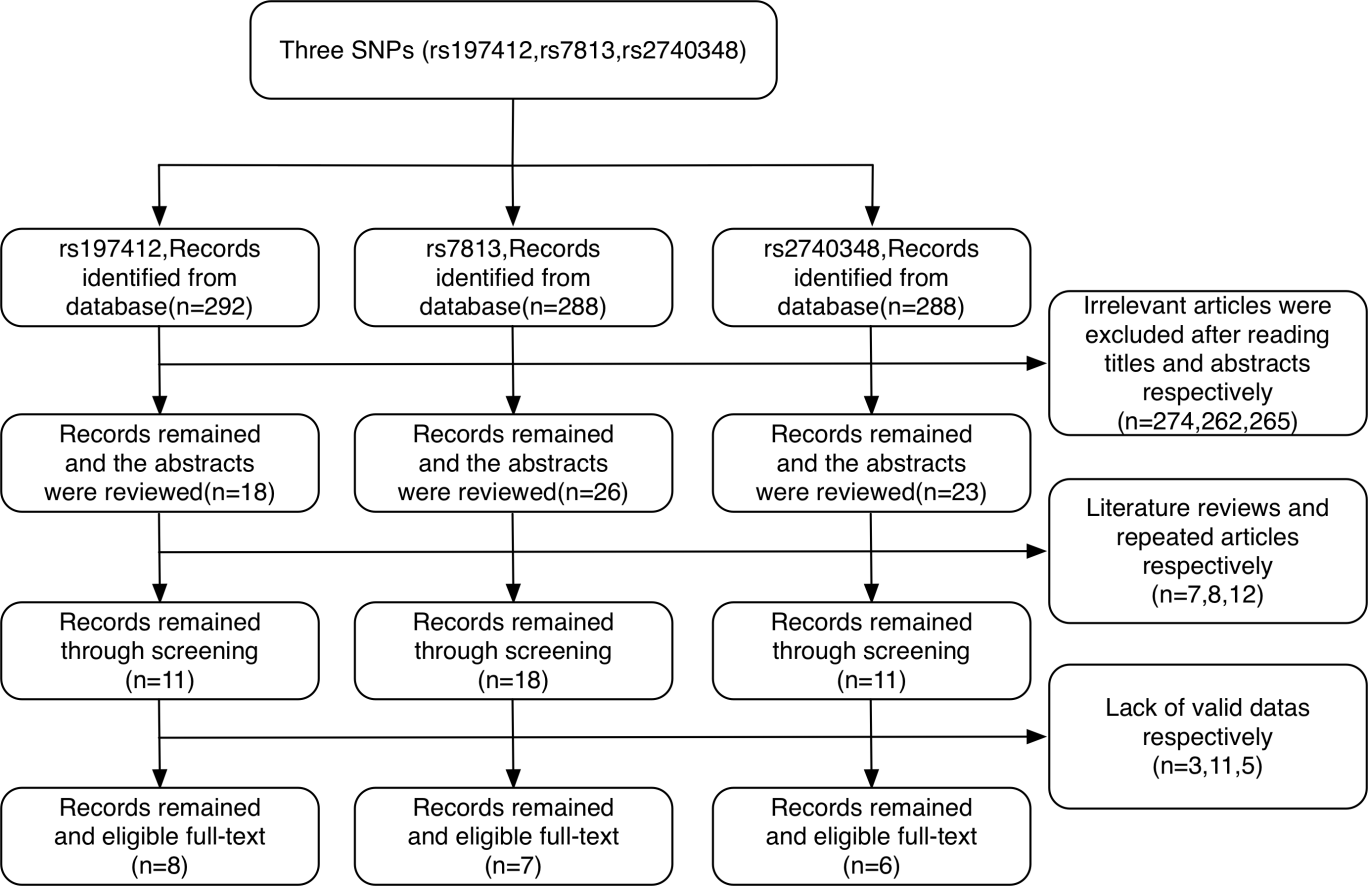
Flowchart for the identification of studies included in the meta-analysis.

As shown in the flow chart (Fig. 2), 292, 288 and 288 articles were collected from the Web of Science, PubMed and CNKI online databases, respectively. Of these, 274, 262 and 265 articles, respectively, were excluded based on examination of the title and abstract for the rs197412, rs7813 and rs2740348 SNPs. We carefully excluded 7, 8 and 12 articles, respectively, that were either literature reviews or repeated articles in sequence. Finally, 22 articles were considered for meta-analysis; eight articles (Chan, 2011; Horikawa et al., 2008; Jiang et al., 2013; Kim et al., 2010; Li, 2013; Roy et al., 2014; Xie et al., 2015; Zhao et al., 2015) were relevant to rs197412, seven articles (Horikawa et al., 2008; Kim et al., 2010; Liang et al., 2010; Liu et al., 2012b; Sung et al., 2011; Yang et al., 2008) (2,588 cases and 2,549 controls) to rs7813 and six articles (Horikawa et al., 2008; Kim et al., 2010; Liu et al., 2012a; Xie et al., 2015; Ye et al., 2008; Zhao et al., 2015) (1,314 cases and 1,244 controls) to rs2740348.

**Table 1:** Characteristics of enrolled studies for rs7813.

Author name	Year	Country	Diseases	Ethnicity	Genotyping methods	Sample size		Case genotype			Control genotype			HWE of Control	Quality
						Case	Control	TT	TC	CC	TT	TC	CC		
Jiaming Liu	2013	China	Prostate cancer	Asian	HRM method	300	242	192	98	10	144	81	17	0.2362	7
Hushan Yang	2008	American	Bladder cancer	Caucasian	SNPlex	736	736	225	381	130	222	352	162	0.3145	8
Yohei Horikawa	2008	American	Renal cell carcinoma	Caucasian	SNPlex	277	278	96	129	52	75	143	60	0.5962	8
Yuanqing Ye	2008	American	Esophageal cancer	Caucasian	SNPlex	280	278	91	137	52	84	138	56	0.9604	7
Dong Liang	2010	American	Ovarian cancer	Caucasian	Illumina	339	349	123	162	54	93	174	82	0.9721	7
Hyuna Sung	2011	Korea	Breast cancer	Asian	TaqMan	558	567	236	254	68	218	267	82	0.1428	8
Jong-Sik Kim	2010	Korea	Lung cancer	Asian	Spectrometry- based	98	99	42	45	11	47	40	12	0.4466	7

**Notes.**

Abbreviations: HRM method high resolution melting method HWE Hardy-Weinberg equilibrium

**Table 2:** Characteristics of enrolled studies for rs2740348.

Author name	Year	Country	Diseases	Ethnicity	Genotyping methods	Sample size		Case genotype		Control genotype		Quality
						Case	Control	GG	GC + CC	GG	GG + CC	
Jiaming Liu	2013	China	Prostate cancer	Asian	HRM method	300	244	246	54	182	62	7
Yohei Horikawa	2008	American	Renal cell carcinoma	Caucasian	SNPlex	276	278	192	84	168	110	8
Ying Xie	2015	China	Gastric cancer	Asian	PCR-LDR	137	144	110	27	115	29	7
Yuanqing Ye	2008	American	Esophageal cancer	Caucasian	SNPlex	346	346	238	108	238	108	7
Yufei Zhao	2015	China	Colorectal cancer	Asian	PCR-LDR	163	142	128	35	114	28	7
Jong-Sik Kim	2010	Korea	Lung cancer	Asian	Spectrometry-based	92	90	74	18	71	19	7

**Notes.**

Abbreviations: HRM method high resolution melting method

**Figure 3:**
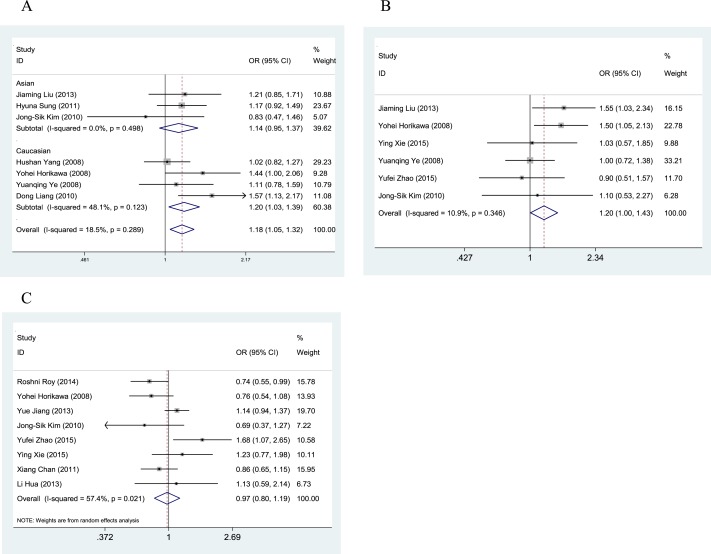
Forest plots of the relationship between cancer and Gemin4 rs7813 (A), Gemin4 rs2740348 (B) and Gemin3 rs197412 (C).

The main characteristics and results of the eligible studies are summarized in Tables 1 and 2. In the present analysis, the results of the meta-analysis of the Gemin4 rs7813 SNP revealed increased cancer risk for TT relative to TC + CC (OR = 1.18, 95% CI [1.05–1.32], *Z*-score = 2.75, *P*-value = 0.006) (Fig. 3A). In a subgroup analysis by ethnicity, the pooled OR of Asians was not positively associated with cancer risk (OR_Asian_ = 1.14, 95% CI [0.95–1.37]). However, in the Caucasian subgroup, the pooled OR was positively associated with cancer risk (OR_Caucasian_ = 1.20, 95% CI [1.03–1.39]). A fixed-effects model was used to evaluate both the Asian and Caucasian subgroups according to study heterogeneity (*I*^2^ < 50). The results of the meta-analysis of the Gemin4 rs2740348 SNP revealed increased cancer risk for GG relative to GC + CC (OR = 1.20, 95% CI [1.00–1.43], *Z*-score = 2.01, *P*-value = 0.044) (Fig. 3B). Due to the limited literature data included in this meta-analysis, we did not perform subgroup analysis on these data. The results of the meta-analysis of the Gemin3 rs197412 SNP showed no significant difference in cancer risk for TT relative to TC + CC (OR = 0.97, 95% CI [0.80–1.19], *Z*-score = 0.25, *P*-value = 0.799) (Fig. 3C).

**Figure 4:**
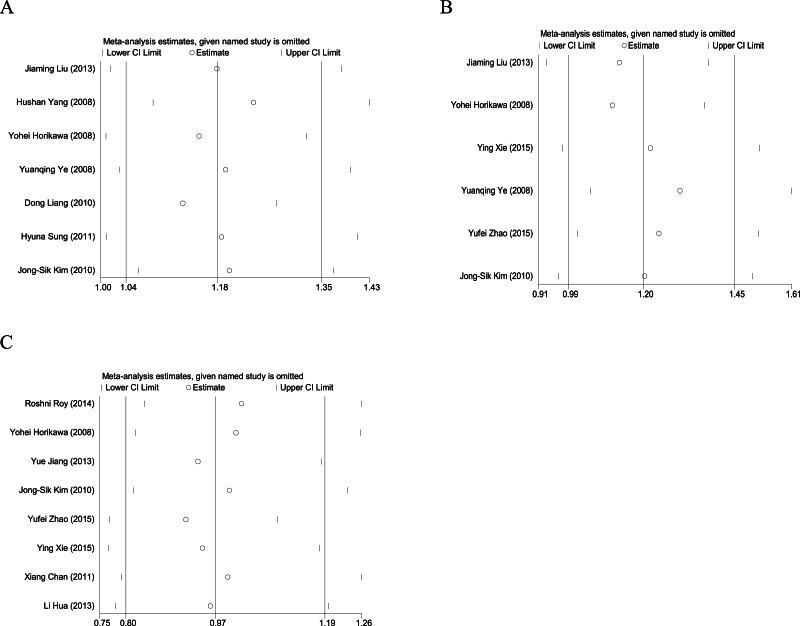
Sensitivity analysis of Gemin4 rs7813 (A), rs2740348 (B) and Gemin3 rs197412 (C).

**Figure 5:**
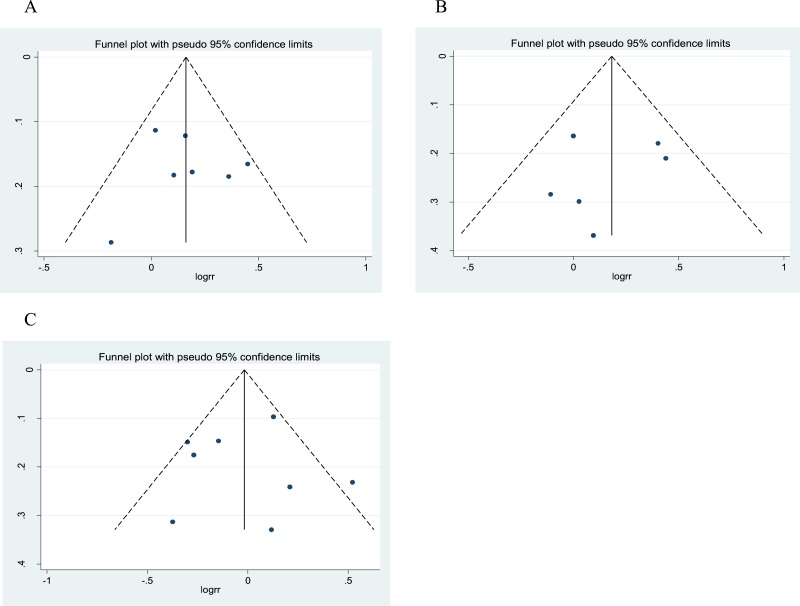
Funnel plot for publication bias analysis of Gemin4 rs7813 (A), rs2740348 (B) and Gemin3 rs197412 (C).

We chose a fixed-effects model to examine the data for rs7813 based on *I*^2^ = 18.5% and the *P*-value of a *Q* test for heterogeneity (Ph) = 0.289. Sensitivity analysis revealed that the pooled ORs were not changed by removing any single study (Fig. 4A). We chose a fixed-effects model to examine the data for rs2740348 based on *I*^2^ = 10.9% and Ph = 0.346. Sensitivity analysis showed that the pooled ORs were not changed by removing any single study (Horikawa et al., 2008; Liu et al., 2012a) (Fig. 4B). We chose a random-effects model to examine the data for rs197412 based on *I*^2^ = 57.4% and Ph = 0.021. Sensitivity analysis revealed that the pooled ORs were not changed by removing any single study (Fig. 4C).

In the funnel plot analysis of rs7813 (Fig. 5A), neither Begg’s funnel plot nor Egger’s test (*P* = 0.849) showed any evidence of publication bias. In the funnel plot analysis of rs2740348 (Fig. 5B), neither Begg’s funnel plot nor Egger’s test (*P* = 0.612) showed any evidence of publication bias. In the funnel plot analysis of rs197412 (Fig. 5C), neither Begg’s funnel plot nor Egger’s test (*P* = 0.920) showed any evidence of publication bias.

Our results suggest that the Gemin4 rs7813 T > C and rs2740348 G > C polymorphisms are associated with cancer susceptibility.

## Discussion

As mentioned previously, miRNAs are emerging as critical regulators of gene expression, as they have been shown to modulate approximately 1/3 of the human genome (Salzman & Weidhaas, 2013). The relationship between miRNAs and cancer has been reported in a host of previous studies (Slaby et al., 2012). We found that some reviews did not provide quantitative information needed for our study. However, this mystery of the diverse expression of miRNAs has not yet been completely solved. Our work investigated the significance of the relationship between Gemin4 polymorphisms and tumorigenesis risk. The goal of our research was to explain the precise mechanisms underlying the distinct expression of miRNAs and to determine the relevance of miRNA biosynthesis genes to cancer susceptibility.

In our meta-analyses, we found that the Gemin4 rs7813 and rs2740348 SNPs were significantly associated with the risk of cancer. Mourelatos et al. (2002) found that the Gemin3 and Gemin4 proteins are present in a 15S ribonucleoprotein complex containing eIF2C, which is pivotal for miRNA processing. Many studies (Esquela-Kerscher & Slack, 2006; Hutvágner & Zamore, 2002; Murashov et al., 2007) have suggested that the interaction of Gemin proteins and key components of the RNA-induced-silencing complex (RISC) could lead to the degradation of target mRNAs.

To date, no meta analysis has evaluated the relationship between polymorphisms in Gemin3 or Gemin4 and cancer risk. Our study selected seven articles, with a pooled total of 2,588 cases and 2,549 controls, relevant to the relationship between the rs7813 SNP and cancer risk, and we found a significant increase in cancer risk for TT relative to TC + CC (TT vs. TC + CC, OR = 1.18, 95% CI [1.05–1.32]. In addition, this association was significant in the Caucasian subgroup (OR = 1.20, 95% CI [1.03–1.39]). The results regarding the Gemin4 rs2740348 SNP were controversial. For this analysis, 6 articles were included, and the pooled OR was a critical value (GG vs. GC + CC, OR = 95% CI [1.00–1.43]). However, we did not conclude that this SNP may increase the incidence of cancer. Had we collected more related studies and a larger sample size, our data would have been more convincing. The *P*-values and *Z*-scores of meta-analyses are widely used to evaluate low-frequency and rare variants. In our study, the *P*-values for rs7813 (*P* = 0.006) (*P* = 0.007) and rs2740348 (*P* = 0.044) enhanced the reliability of our results. We did not show the results for HWE in Table 2 because all collected data were related to the GG and GC + CC genotypes, and HWE could not be calculated. However, the authors of these studies indicated that all gene polymorphisms of the control group conformed to HWE.

Next, we investigated the Gemin3 rs197412 SNP, which is a key indicator of renal cell carcinoma. In this meta-analysis, however, rs197412 was not associated with increased cancer risk. In addition, we searched for articles related to the Gemin3 rs197414 and rs197338 SNPs, and the Gemin4 rs3744741 and rs4968104 SNPs. However, we identified fewer than five articles, which was insufficient for us to evaluate the precise relationship between these SNPs and cancer risk. However, we found several articles that reported an association between Gemin polymorphisms and several chronic diseases, such as hepatitis B (Shang et al., 2014). The relationship between Gemin and these chronic diseases, as well as cancer, should be investigated further.

The method of selection of a fixed-effects or random-effects model did not follow the gold standard. Generally, most recent studies have used one or a combination of the traditional fixed-effects or random-effects models. However, some drawbacks regarding combining meta-analysis results from multi-ethnic genome-wide association studies (GWASs) persist (Li & Keating, 2014). These methods overlook transethnic effects to obtain population-wide associations. The degree of heterogeneity also impacts standard error, thus affecting statistical values, and this effect results in some bias when combining meta-analysis results from multi-ethnic GWASs (Wang et al., 2013). Higgins’s (*I*^2^) test was used to evaluate study heterogeneity. In our study, the *I*^2^ values were small; thus, the effect of ethnicity was small. Additional subgroup analyses by ethnicity would supplement our results.

With respect to methodological quality, the greatest limitation of our study was the relatively small population size, which may lead to type II error. Each polymorphism can produce discrepant effects between different genetic backgrounds (Lin et al., 2007). Alternatively, the differences between studies may be due to differences in cancer types, which have different etiologies and utilize distinct carcinogenesis pathways. Furthermore, the sample size can affect the accuracy of the results, and the presence of unknown confounding factors must be considered (Kim et al., 2010). Although a subgroup analysis by ethnicity was conducted, no study had examined the African population. Thus, the results of our study are incomplete.

In conclusion, our meta-analyses provided statistical evidence that the Gemin4 rs7813 and rs2740348 SNPs can predict cancer prognosis. However, we need to perform further research on the association of the rs2740348 SNP with cancer risk to provide more powerful evidence of a true association. We hope that the results of our study will aid in identifying the roles of miRNAs in cancer prevention and control.

## Supplemental Information

10.7717/peerj.1724/supp-1 Supplemental Information 1 PRISMA 2009 Checklist

10.7717/peerj.1724/supp-2 Supplemental Information 2 S3 rs197412 Raw data

10.7717/peerj.1724/supp-3 Supplemental Information 3 Raw data

## Additional Information and Declarations

### Data Availability

The following information was supplied regarding data availability: Data in this article came from PubMed, Web of Science and the China National Knowledge Infrastructure (CNKI) online databases.
