# Supplementary material for: Genetic variants in the MicroRNA biosynthetic pathway Gemin3 and Gemin4 are associated with a risk of cancer: a meta-analysis
Source: PeerJ. 2016 Mar 15;4:e1724. doi: 10.7717/peerj.1724 (PMC4806601; doi:10.7717/peerj.1724)
Supplement: Supplemental Information 2 [file peerj-04-1724-s002.docx]

| Author Name | Year | Diseases | Sample size | | Case genotype | | | Control genotype | | |
| --- | --- | --- | --- | --- | --- | --- | --- | --- | --- | --- |
|  |  |  | Case | Control | TT | TC+CC | TT | | TC+CC |  |
| Roshni Roy | 2014 | oral cancer | 447 | 450 | 113 | 334 | 141 | | 309 |  |
| Yohei Horikawa | 2008 | renal cell carcinoma | 277 | 278 | 97 | 180 | 115 | | 163 |  |
| Yue Jiang | 2013 | breast cancer | 853 | 886 | 380 | 473 | 367 | | 519 |  |
| Jong-Sik Kim | 2010 | lung cancer | 92 | 90 | 28 | 64 | 35 | | 55 |  |
| Yufei Zhao | 2015 | colorectal cancer | 163 | 142 | 90 | 73 | 60 | | 82 |  |
| Ying Xie | 2015 | gastric cancer | 137 | 142 | 65 | 72 | 60 | | 82 |  |
| Xiang Chan | 2011 | breast cancer | 354 | 473 | 122 | 232 | 179 | | 294 |  |
| Li Huan | 2013 | lymphoma | 69 | 96 | 45 | 24 | 60 | | 36 |  |

Rs197412 raw data
