# Supplementary material for: Genetic variants in the MicroRNA biosynthetic pathway Gemin3 and Gemin4 are associated with a risk of cancer: a meta-analysis
Source: PeerJ. 2016 Mar 15;4:e1724. doi: 10.7717/peerj.1724 (PMC4806601; doi:10.7717/peerj.1724)
Supplement: Supplemental Information 3 [file peerj-04-1724-s003.docx]

Table 1. Characteristics of enrolled studies for rs7813.

| Author Name | Year | Country | Diseases | Ethnicity | Genotyping methods | Sample size | | Case genotype | | | Control genotype | | | HWE of | Quality |
| --- | --- | --- | --- | --- | --- | --- | --- | --- | --- | --- | --- | --- | --- | --- | --- |
|  |  |  |  |  |  | Case | Control | TT | TC | CC | TT | TC | CC | control |  |
| Jiaming Liu | 2013 | China | prostate cancer | Asian | HRM method | 300 | 242 | 192 | 98 | 10 | 144 | 81 | 17 | 0.2362 | 7 |
| Hushan Yang | 2008 | American | bladder cancer | Caucasian | SNPlex | 736 | 736 | 225 | 381 | 130 | 222 | 352 | 162 | 0.3145 | 8 |
| Yohei Horikawa | 2008 | American | renal cell carcinoma | Caucasian | SNPlex | 277 | 278 | 96 | 129 | 52 | 75 | 143 | 60 | 0.5962 | 8 |
| Yuanqing Ye | 2008 | American | esophageal cancer | Caucasian | SNPlex | 280 | 278 | 91 | 137 | 52 | 84 | 138 | 56 | 0.9604 | 7 |
| Dong Liang | 2010 | American | ovarian cancer | Caucasian | Illumina | 339 | 349 | 123 | 162 | 54 | 93 | 174 | 82 | 0.9721 | 7 |
| Hyuna Sung | 2011 | Korea | breast cancer | Asian | TaqMan | 558 | 567 | 236 | 254 | 68 | 218 | 267 | 82 | 0.1428 | 8 |
| Yuxiong Weng | 2015 | China | osteosarcoma | Asian | iPLEX system | 143 | 143 | 72 | 59 | 12 | 70 | 60 | 13 | 0.9778 | 7 |
| Jong-Sik Kim | 2010 | Korea | lung cancer | Asian | spectrometry-based | 98 | 99 | 42 | 45 | 11 | 47 | 40 | 12 | 0.4466 | 7 |

Abbreviations: HRM method, high resolution melting method; HWE, Hardy-Weinberg equilibrium.

Table 2. Characteristics of enrolled studies for rs2740348.

| Author Name | Year | Country | Diseases | Ethnicity | Genotyping methods | Sample size | | Case genotype | | Control genotype | | Quality |
| --- | --- | --- | --- | --- | --- | --- | --- | --- | --- | --- | --- | --- |
|  |  |  |  |  |  | Case | Control | GG | GC+CC | GG | GG+CC |  |
| Jiaming Liu | 2013 | China | prostate cancer | Asian | HRM method | 300 | 244 | 246 | 54 | 182 | 62 | 7 |
| Yohei Horikawa | 2008 | American | renal cell carcinoma | Caucasian | SNPlex | 276 | 278 | 192 | 84 | 168 | 110 | 8 |
| Ying Xie | 2015 | China | Gastric cancer | Asian | PCR-LDR | 137 | 144 | 110 | 27 | 115 | 29 | 7 |
| Yuanqing Ye | 2008 | American | esophageal cancer | Caucasian | SNPlex | 346 | 346 | 238 | 108 | 238 | 108 | 7 |
| Yufei Zhao | 2015 | China | colorectal cancer | Asian | PCR-LDR | 163 | 142 | 128 | 35 | 114 | 28 | 7 |
| Jong-Sik Kim | 2010 | Korea | lung cancer | Asian | spectrometry-based | 92 | 90 | 74 | 18 | 71 | 19 | 7 |

Abbreviations: HRM method, high resolution melting method.
